# Kinome profiling of myxoid liposarcoma reveals NF-kappaB-pathway kinase activity and Casein Kinase II inhibition as a potential treatment option

**DOI:** 10.1186/1476-4598-9-257

**Published:** 2010-09-23

**Authors:** Stefan M Willems, Yvonne M Schrage, Inge H Briaire-de Bruijn, Karoly Szuhai, Pancras CW Hogendoorn, Judith VMG Bovée

**Affiliations:** 1 Department of Pathology, Leiden University Medical Center, Leiden, The Netherlands; 2 Department of Molecular Cell Biology, Leiden University Medical Center, Leiden, The Netherlands

## Abstract

**Background:**

Myxoid liposarcoma is a relatively common malignant soft tissue tumor, characterized by a (12;16) translocation resulting in a FUS-DDIT3 fusion gene playing a pivotal role in its tumorigenesis. Treatment options in patients with inoperable or metastatic myxoid liposarcoma are relatively poor though being developed and new hope is growing.

**Results:**

Using kinome profiling and subsequent pathway analysis in two cell lines and four primary cultures of myxoid liposarcomas, all of which demonstrated a FUS-DDIT3 fusion gene including one new fusion type, we aimed at identifying new molecular targets for systemic treatment. Protein phosphorylation by activated kinases was verified by Western Blot and cell viability was measured before and after treatment of the myxoid liposarcoma cells with kinase inhibitors. We found kinases associated with the atypical nuclear factor-kappaB and Src pathways to be the most active in myxoid liposarcoma. Inhibition of Src by the small molecule tyrosine kinase inhibitor dasatinib showed only a mild effect on cell viability of myxoid liposarcoma cells. In contrast, inhibition of the nuclear factor-kappaB pathway, which is regulated by the FUS-DDIT3 fusion product, in myxoid liposarcoma cells using casein kinase 2 inhibitor 4,5,6,7-tetrabromobenzotriazole (TBB) showed a significant decrease in cell viability, decreased phosphorylation of nuclear factor-kappaB pathway proteins, and caspase 3 mediated apoptosis. Combination of dasatinib and TBB showed an enhanced effect.

**Conclusion:**

Kinases associated with activation of the atypical nuclear factor-kappaB and the Src pathways are the most active in myxoid liposarcoma *in vitro *and inhibition of nuclear factor-kappaB pathway activation by inhibiting casein kinase 2 using TBB, of which the effect is enhanced by Src inhibition using dasatinib, offers new potential therapeutic strategies for myxoid liposarcoma patients with advanced disease.

## Background

Myxoid liposarcoma accounts for 40% of all liposarcomas and occurs most commonly in the extremities [1]. In about 95% of cases, myxoid liposarcoma is cytogenetically characterized by t(12;16)(q13;p11), creating a chimerical FUS/DDIT3 gene which has been thought to play a pivotal role in its tumourigenesis [2-4]. The cornerstone of curative treatment for myxoid liposarcoma is surgery with an overall 10 years survival of 80%. Prognosis is mainly determined by the percentage of round cell component of the tumor. Myxoid liposarcoma with more than 5% round cell component are defined as high-grade and prone to metastasis[5]. Treatment options for patients with inoperable or metastatic disease are relatively poor, though trials with new drugs reveal good perspectives for the future [6,7]. Therefore, clinical trials to test and validate new treatment options for liposarcoma subtypes (such as myxoid liposarcoma) are necessary[6]. Nowadays, (neo) adjuvant chemotherapy of liposarcoma patients is limited with only ifosfamide and anthracyclins showing 20-40% response rates in untreated patients[8]. Trabectedin (Yondelis, ET 743) is a novel chemotherapeutic agent derived from the marine tunicate *Ecteinascidia turbinate*. By binding to the DNA minor groove, ET-743 forms covalent adducts with the N2-position of guanine through its carbinolamine moiety. As a result, the minor groove bends toward the major groove. The cytotoxic activity of ET-743 is largely based on its interaction with nucleoside excision repair machinery, as well as through the induction of double strand breaks[9-11]. Phase I and II studies showed promising results in myxoid liposarcoma patients with advanced disease though recent studies reported an increasing number of side effects[12,13]. During the last years, tumor specific targeted therapy has shown to be effective in many cancers, including sarcomas. Especially kinase inhibitors are an emerging class of small molecule inhibitors that target unique kinase conformational forms and binding sites[14]. Notable advantages are higher specificity and generally more manageable and reversible side effects [15]. This necessitates the study of separate soft tissue tumour entities[7]. In the present study, we explored the activated pathways in myxoid liposarcoma cells using kinome profiling to find new treatment possibilities. Kinases phosphorylate tyrosine, threonine or serine residues on proteins, thereby serving as a switch to (in) activate pathways involved in cell cycle, cell survival and differentiation. Moreover, kinases are promising targets for anti-cancer therapy as they do not require new protein synthesis, therefore act rapidly and are also promising in slow-cycling tumors [16,17].

Data on activated pathways in myxoid liposarcoma are sparse[18,19]. By using a kinase substrate specific protein array chip combining 1024 different kinase substrates, we identified kinases associated with Src and NF-kappaB pathways to be active in myxoid liposarcoma. NF-kappaB is an inducible cellular transcription factor that regulates a variety of cellular genes, including those involved in immune regulation, inflammation, cell survival and cell proliferation. Hereby, active NF-kappaB plays a pivotal role in tumorigenesis and increased expression of the phosphorylated NF-kappaB protein is found in many tumors[20,21]. We showed that in myxoid liposarcoma cells, inhibition of kinases associated with the NF-kappaB pathway (by TBB) resulted in decreased viability and that this effect was enhanced by Src-inhibitor dasatinib. These results show that targeting NF-kappaB pathway might be a potential treatment option in myxoid liposarcoma patients with advanced disease.

## Results

### Molecular and cytogenetic analysis

**Figure 1:**
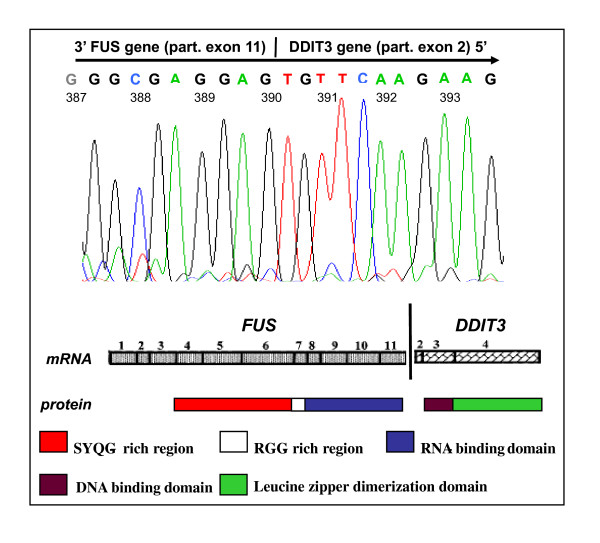
**Exon 11 of the FUS gene is fused to exon 2 of the DDIT3 gene**. The corresponding chimeric protein retains the RNA binding domain of the FUS protein.

FISH of the primary myxoid liposaromas showed the tumor specific t(12;16) in three out of four cases (table 1). All four primary cultures showed the FUS/DDIT3 fusion transcripts[22]. Case L1187 showed a 1033 bp long fusion transcript involving exon 11 of the FUS and exon 2 of the DDIT3 gene, which has not been reported previously (figure 1). This chimera includes the RNA-binding domain (exon 8-11) of the FUS gene as in fusion type 8, which is absent in the other fusion types. This new FUS/DDIT3 fusion type was deposited in GenBank (GenBank accession number GU933437). COBRA-FISH of both myxoid liposarcoma cell lines showed the myxoid liposarcoma specific t(12;16) translocation. The precise karyotype of 402-91 was: 46, X, der(Y)t(Y;19)(q11;p11), t(1;7)(p12;p12), der(8)t(8;21)(p11;p11)[7], der(8)t(8;9)(p11;p11)[7], del(8)(p11)[4], del(10)(p11), t(12;16)(q13;p11), del(18)(p11), -19,+20, -21[7][cp20], several additional, non-clonal rearrangements involving chromosomes 4, 5, 6 and 8 with various partner chromosomes. The precise karyotype of 1765-92 was 90-99, XX, der(1)inv(1)(p32q31)t(1;10)(p33;p12), der(1)inv(1)(p32q31)t(1;10) (p33;p12), -1, del(2)(p11), -3, +5, der(6)t(4;6)(4q, 6q), der(6)t(6;10)(p;q), +der(6)t(6;10) (p;q), der(8)t(3;8), i(8)(q10), +i(8)(q10), +9, der(10)t(1;10)(1p32, p12), der(10)t(1;10) (1p32, p12), -10, +11, t(12;16)(q13;p11), t(12;16)(q13;p11), -13, der(13)t(6;13)(q;q), +14, +15, +18, +20, +20 [cp20].

**Table 1:** Clinicopathological and genetic data of myxoid liposarcoma samples

	Sample ID	Type	Gender	Age	P/R/M	Location	FUS/DDIT3 transcript size	FUS/DDIT3 transcript type	(COBRA) FISH
1	L1187	primary culture	F	20	P	left hamstrings	1033 bp	X	t(12;16)
2	L1357	primary culture	M	50	P	left hamstrings	654 bp	I	t(12;16)
3	L1434	primary culture	F	43	P	right hamstrings	654 bp	I	t(12;16)
4	L2187	primary culture	F	42	P	left thigh, subcutaneously	378 bp	II	N/A
5	402-91	cell line	M	unknown	unknown	unknown	unknown	N/A	t(12;16)
6	1765-92	cell line	unknown	unknown	unknown	unknown	unknown	N/A	t(12;16)

Primary cultures of samples L1187, L1357, L1434 and L2187 were obtained from fresh tumors. Tumors L1187 and L1357 were high grade (> 5% round cell component), whereas L1434 and L2187 were low-grade (< 5% round cell component). These differences in grade however were not reflected in growth rate of the primary cultures (doubling times of primary cultures were all ~ 4 days; doubling times of cell lines ~ 2 days).

### Identification of active kinases and pathways

A list of phosphorylated targets and their corresponding active kinases was created by kinome profiling of two cell lines and four primary cultures of myxoid liposarcoma. Average spot intensity and target frequency of the top 100 phosphorylated substrates revealed the most activated kinases in myxoid liposarcoma (table 2). Both in myxoid liposarcoma cell lines as well as in primary cultures, casein kinase 2, alpha 1 (ck2a1), lymphocyte-specific protein tyrosine kinase (lck), fyn oncogene related to SRC (fyn), Gardner-Rasheed feline sarcoma viral (v-fgr) oncogene homolog (fgr), v-yes-1 Yamaguchi sarcoma viral oncogene homolog (yes), calcium/calmodulin-dependent protein kinase II beta (camk2b) and protein kinase, cAMP-dependent, catalytic, alpha (prkaca) were most activated (table 2). There were no clear differences between the cell lines and the primary cultures. The specificity of the list of substrates for myxoid liposarcomas was verified by comparing the intensity of the signals with those for normal MSCs which served as a normal control for this tumor type, using Limma (additional file 1). Specificity of the activated kinases in this type of cancer (i.e. myxoid liposarcoma) was additionally verified by comparison with the same analysis in four colorectal carcinoma cell lines and thirteen chondrosarcoma cell lines and cultures using Limma, which revealed a different list of substrates and kinases[16]. Pathway analysis based on the most active kinases (table 3) identified kinases associated with NF-kappaB pathway (ck2a1, fgr, inhibitor of kappa light polypeptide gene enhancer in B-cells, kinase (ikk), protein kinase RNA-activated (pkr), v-akt murine thymoma viral oncogene homolog (akt), NF-kappa-beta-inducing kinase (nik), mitogen-activated protein kinase kinase kinase 3 (mekk3) and focal adhesion kinase 1 (fak1) to be most activated. Also kinases associated with Src-pathway (lck, fyn) were highly active. In addition, retinoic acid receptor pathway (RAR) and peroxisome proliferator-activated receptor (PPAR) activation pathway were found. The top 5 of activated pathways was identical in cell lines and primary cultures. Results of the analysis leaving out all cell cycle related kinases (27% of all kinases detectable), which might be artificially upregulated due to cell culturing, and results of analysis after starvation of the cell lines are shown in table 3.

**Table 2:** Top 100 activated kinases and targeted drugs in myxoid liposarcoma cell lines and primary cultures.

	Intensity	Kinase	Number of hits	Description	Drugs
1	7965,340	CK2	9	Casein kinase 2, alpha 1	4,5,6,7-tetrabromobenzotriazole
2	5932,666	LCK	3	Lymphocyte-specific protein tyrosine kinase	dasatinib
3	5932,666	FYN	2	FYN oncogene related to SRC, FGR, YES	dasatinib
4	4716,473	CAMK2B	5	Calcium/calmodulin-dependent protein kinase II beta	
5	3998,331	PRKACA	8	Protein kinase, cAMP-dependent, catalytic, alpha	
6	3922,920	MAPK1	4	Mitogen-activated protein kinase 1 [	
7	3922,920	KIT	1	V-kit Hardy-Zuckerman 4 feline sarcoma viral oncogene homolog	dasatinib, sunitinib ao
8	3402,973	CSNK1A1	1	Casein kinase 1, alpha 1	
9	3402,973	CIB	2	Calcium and integrin binding family	
10	3317,951	GSK3	2	Glycogen synthase kinase 3	
11	3144,082	LYN	1	V-yes-1 Yamaguchi sarcoma viral related oncogene homolog	
12	3144,082	BTK	1	Bruton agammaglobulinemia tyrosine kinase	
13	3114,907	PKC	19	Protein kinase C	
14	3057,647	AKT1	3	V-akt murine thymoma viral oncogene homolog 1	enzastaurin
15	3033,443	PKM2	1	Pyruvate kinase, muscle	
16	2928,117	CAMK1	1	Calcium/calmodulin-dependent protein kinase I	
17	2893,922	CHEK1	1	CHK1 checkpoint homolog	
18	2893,922	CHEK2	2	CHK2 checkpoint homolog	
19	2893,922	PLK3	2	Polo-like kinase 3	
20	2890,210	TTN	1	Titin	
21	2712,053	ABL	2	Abelson murine leukemia viral (v-abl) oncogene homolog	imatinib, temozolomide
22	2596,825	INSR	1	Insulin receptor	lispro, aspart, glargine
23	2596,825	EGFR	2	Epidermal growth factor receptor	cetuximab, canertinib ao
24	2596,825	MET	1	Met proto-oncogene	
25	2483,173	SRC	6	V-src sarcoma (Schmidt-Ruppin A-2) viral oncogene homolog	
26	2443,714	RPS6	4	Ribosomal protein S6	
27	2382,003	CK	1	Choline kinase	
28	2314,823	MAP2K3	1	Mitogen-activated protein kinase kinase 3	
29	2294,228	GRK1	2	G protein-coupled receptor kinase 1	
31	2216,637	JAK1	1	Janus kinase 1	
32	2214,443	MAPKAPK2	1	Mitogen-activated protein kinase-activated protein kinase 2	
33	2179,345	ALK	1	Anaplastic lymphoma receptor tyrosine kinase	
34	2052,700	ATM	2	Ataxia telangiectasia mutated	
35	2040,371	PKN1	2	Protein kinase N1	
36	1913,813	PDGFRB	1	Platelet-derived growth factor receptor, beta polypeptide	dasatinib, sunitib ao
37	1870,956	CDK2	1	Cyclin-dependent kinase 2	BMS-387032, flavopiridol
38	1849,628	CCRK	1	Cell cycle related kinase	
39	1806,824	CDC2	1	Cell division cycle 2, G1 to S and G2 to M	flavopiridol

Top list of kinases was based on the intensity of incorporated radioactively labeled phosphorus, corresponding with kinase activity. The number of hits corresponds to the number of substrates to be phosphorylated by a specific kinase, not necessarily associated with kinase activity as substrates were not equally covered by the different kinases.

**Table 3:** Top lists of activated kinases and pathways in different conditions

	top five activated kinases	top activated signalling pathway
normal medium condition (including all kinases in the analysis)	ck2a1	NF-kappaB
	lck	Src
	fyn	RAR
	fgr	PPAR
	yes	

normal medium condition (leaving out cell cycle related kinases in the analysis)	ck2a1	NF-kappaB
	lck	Src
	fyn	RAR
	camk2b	PPAR
	prkcd	

starved medium condition	mapk14	NF-kappaB
	ck2a1	RAR
	akt1	p53
	egfr	G1/S transition of the cell cycle
	erbb2	oxidative stress response

Whereas activated kinases differed between cells grown in normal medium (RMPI supplemented with 10% inactivated calf serum), top five of activated signaling pathways were identical. Results were identical for cell lines and primary cultures. Cell lines cultured in starved medium conditions revealed a different top list of both kinases and activated signaling pathways. However, in all three different conditions, NF-kappaB was the most activated signaling pathway identified.

prkcd: protein kinase C, delta; mapk14: mitogen-activated protein kinase 14; egfr: epidermal growth factor receptor; erbb2: v-erb-b2 erythroblastic leukemia viral oncogene homolog 2

### Verification of kinome profiling

**Figure 2:**
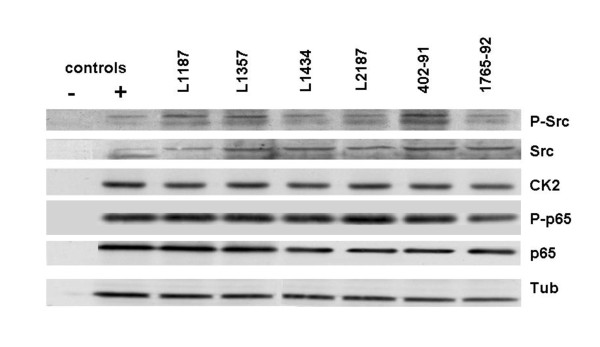
**Immunoblotting was used for verification of the results obtained by Pepchip analysis**. Band heights were all corresponding to the manufacturer's datasheets (P-Src: 2 bands between 56-61 kDa, Src: 60 kDa, Casein Kinase 2: 42 kDa, P-p65: 65 kDa and p65: 65 kDa). Both cell lines and primary cultures showed phosphorylation of Src and slight variation in amounts of total Src. Casein Kinase 2 and p65 protein were present in all samples in comparable amounts, as is phosphorylated p65, indicating active NF-kappaB signaling.

Western blotting showed that all myxoid liposarcoma samples (both cell lines and primary cultures) expressed comparable amounts of total Src and NF-kappaB p65. Phosphorylation of Src (Y419) was present in all samples (figure 2) confirming activation of Src pathway. Likewise, western blotting showed the presence of ck2a1 and phosphorylated NF-kappaB p65 (S468) in all samples, confirming the results of the IPA analysis that kinases associated with NF-kappaB pathway are active in myxoid liposarcoma cells.

### In vitro targeting of kinases associated with Src and NF-kappaB pathways by dasatinib and TBB

**Figure 3:**
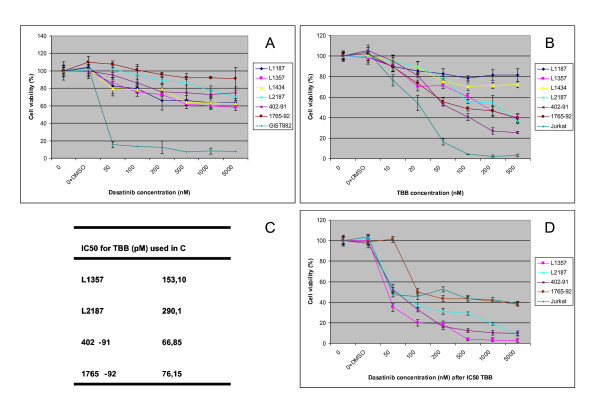
**Effect of dasatinib and TBB treatment on cell viability of myxoid liposarcoma cells.** 3A) Treatment of myxoid liposarcoma cell cultures with dasatinib leads to maximum 20% decrease in viability at the concentrations higher than 200 nM, though this effect is limited as compared to GIST882. 3B) Treatment with TBB shows a 20% decrease at lower concentrations (20 μM) and a maximum decrease of 75% at 200 μM (cell line 402-92) in the majority of cases. Cell viability with IC50 s as depicted in 3C. 3D) Combined treatment with TBB (at IC50 concentration) and dasatinib (concentrations as in 3A). Jurkat cells where susceptible to TBB, but not to dasatinib. Interestingly, the effect of dasatinib when preceded by TBB was significantly more pronounced than dasatinib in monodrug treatment which means that dasatinib and TBB have an enhanced effect. Graphs show data from four representative experiments. Error bars indicate the standard error of the mean.

WST-1 analysis of GIST882 showed a profound decrease in cell viability of up to ~ 80% relative to the DMSO control at even low dosages of Src-inhibitor dasatinib (figure 3). The decrease in cell viability of myxoid liposarcoma cells treated with dasatinib was rather mild as WST-1 analysis of all four cell cultures and 1 out of 2 cell lines showed a maximum decrease in cell viability of 40% at higher doses (figure 3). Cell line 1765-92 did not respond to dasatinib. In contrast, myxoid liposarcoma cells showed a decline of more than 50% in viability after treatment with casein kinase 2-inhibitor TBB in two out of four cultures and in both cell lines. This effect was also observed in Jurkat cells as described (positive control)[23]. There was no relation between the response rate and type of fusion gene. For combination experiments, the two cell lines (402-91 and 1765-92) and the two most sensitive myxoid liposarcoma primary cultures (L1357 and L2187) were treated with both dasatinib and TBB. Combined administration of both drugs led to a dramatic decrease in cell viability and showed an enhanced effect (figure 3D), for instance: L1357 cells show 80% viability at maximum dasatinib dose (5000 nM), whereas viability was only 5% at lower concentration of dasatinib (500 nM) at IC 50 for TBB (figure 3D).

### Dasatinib inhibits phosphorylation of Src but does not cause apoptosis

**Figure 4:**
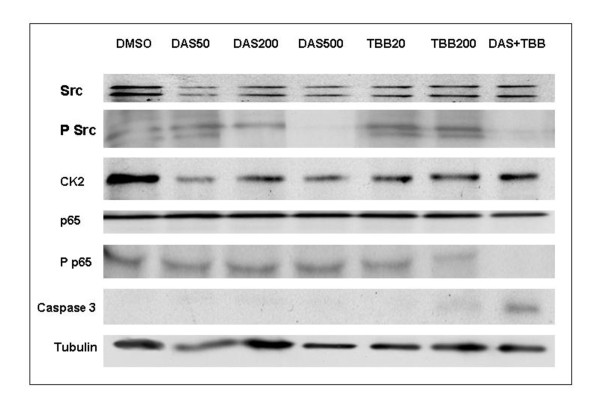
**Effect of dasatinib and TBB treatment on phosphorylation of Src and NF-kappaB related proteins**. Experiments were run in duplicate and showed similar results in two cell lines (402-91 and 1765-92) and primary cultures (L1357 and L2187). Treatment of L1357 with dasatinib did not affect total levels of Src, but gradually decreased P-Src levels at 200 nM to almost absence at 500 nM. There was no effect of dasatinib on total p65 and phosphorylated-p65 levels. Treatment of L1357 with TBB did not affect total levels of p65, but gradually decreased P-p65 levels at 200 μM. TBB treatment had no effect on the levels of total Src and phosphorylated Src. Interestingly, TBB and dasatinib showed enhancement to decrease levels of phosphorylated Src and p65. Strikingly, there was a gradual increase in caspase-3 levels upon treatment with TBB, which was enhanced by combination with dasatinib, suggesting caspase-3 mediated apoptosis underlying the observed decrease in cell viability. Abbreviations: DAS50 = dastinib 50 nM, etc. DAS and TBB = 200 μM dasatinib and IC50 concentration for TBB.

To investigate the effect of dasatinib on Src signalling, a good responsive (60% cell viability at 500 nM; figure 3A) myxoid liposarcoma cell culture (L1357) was treated with 50, 200 and 500 nM of dasatinib for 6 hours. Whereas levels of total Src did not visibly decrease upon dasatinib treatment, a decrease in phosphorylated Src (p-Src) (Y419) was found (figure 4). At a dose of 200 nM dasatinib p-Src staining the lower band faded and at 500 nM both bands disappeared. Interestingly, a similar decrease in p-Src was also observed at 200 nM dasatinib when post-treated with TBB. There was no effect of dasatinib treatment on total NF-kappaB p65 or phosphorylated NF-kappaB p65 and there was no caspase-3 mediated apoptosis, since the level of caspase-3 did not increase upon dasatinib treatment (figure 4).

### TBB inhibits NF-kappaB p65-phosphorylation resulting in caspase-3 mediated apoptosis

To investigate the effect of TBB on kinases associated with NF-kappaB signalling, L1357 was treated with increasing doses for 6 hours. Whereas levels of total NF-kappaB p65 did not decrease upon treatment, a decrease in phosphorylated p65 (p-p65) was found (figure 4). At a dose of 20 μM TBB p-p65 staining slightly started to fade and obviously decreased at 200 μM TBB. Casein Kinase 2 levels of TBB treated samples were lower than the DMSO control, but remained unchanged compared to samples treated with various concentrations TBB or dasatinib, suggesting that TBB does not alter the overall expression of casein Kinase 2, which is in accordance with the literature[24]. TBB treatment had no effect on the levels of total Src and phosphorylated Src. Strikingly, the effect of TBB was increased by pretreatment with dasatinib (figure 4), which was also visible in the viability assay (figure 3D). Moreover, there was a gradual increase in caspase-3 levels upon treatment with TBB, suggesting caspase-3 mediated apoptosis.

## Discussion

Treatment options for myxoid liposarcoma patients with advanced disease are poor. Recently, the chemotherapeutic drug Trabectedin showed promising results in phase I and II trials in advanced disease though adverse effects have also been reported[13,25]. Small molecule targeting, especially with kinase inhibitors, has shown to be effective and more specific in many tumors with less severe side effects than conventional chemotherapeutic agents. To identify new potential treatment options for myxoid liposarcoma patients with advanced disease, we explored the kinome of myxoid liposarcoma cells *in vitro *and performed subsequent pathway analysis.

We previously established the reliability of kinome profiling using Pepchip in untreated versus imatinib treated GIST882 cell line which correctly identified the pathways known to be involved in GIST[16]. Moreover, we previously demonstrated the reliability of our analysis which is based on averaging results of a number of samples to get an impression of the most activated kinases in a series of tumors[16]. By additionally performing the Pepchip experiments in the myxoid liposarcomas cell lines after serum starvation as well as by excluding cell cycle related kinases from the analysis we determined that the detected kinases in the present analysis are indeed tumor specific and not related to the high proliferation rate of the myxoid liposarcoma cell lines. Moreover, by comparing with previously analyzed series of colorectal cancer and chondrosarcoma, as well as by comparing with mesenchymal stem cells we could confirm that the list of kinases was specific for myxoid liposarcomas.

**Figure 5:**
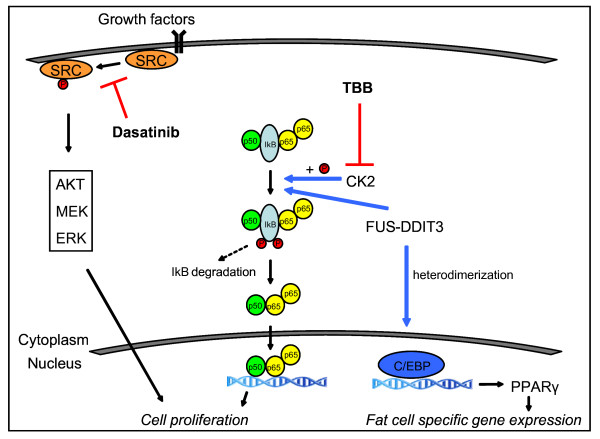
**In myxoid liposarcoma, the FUS/DDIT3 protein has been shown to upregulate the expression of CCAAT/enhancer binding protein (C/EBP) which leads to the transcription of peroxisome proliferator-activated receptors gamma and other genes involved in adipocytic differentiation**. We showed that in myxoid liposarcoma, the atypical NF-kappaB pathway is active. Hereby casein kinase 2 phosphorylates the nuclear factor of kappa light polypeptide gene enhancer in B-cells inhibitor (IkB), which releases from the NF-kappaB p50/p65 complex and gets degraded. The NF-kappaB p50/p65 complex then shuttles into the nucleus were it promotes the transcription of genes involved in cell proliferation. Recent studies showed that the FUS/DDIT3 protein facilitates DNA binding of the NF-kappaB p50/p65 complex in a non-direct manner, probably by interfering with IkB. Also Src pathway is activated in myxoid liposarcoma, which leads through different signaling pathways (AKT, MEK, ERK) to tumor growth and cell survival. This pathway can be inhibited *in vitro *by Src-inhibitor dasatinib.

We could demonstrate activation of the peroxisome proliferator-activated receptor gamma pathway, which could be expected since it has been shown to play a pivotal role in adipocytic differentiation and is regulated by the FUS-DDIT3 fusion product[26-28](Figure 5). The DDIT3 gene encodes a DNA-damage inducible member of the C/EBP family of transcription factors and inhibits adipocytic conversion of preadipocytes[29,30]. Transfection of primary mesenchymal progenitor and human fibrosarcoma cells with the FUS/DDIT3 fusion protein induces a myxoid liposarcoma phenotype[31,32]. Treatment of myxoid liposarcoma cells in *vitro *and *in vivo *with peroxisome proliferator-activated receptors gamma agonists induced terminal differentiation[33], although phase II studies with the peroxisome proliferator-activated receptors gamma agonist Rosiglitasone did not show the antitumor effect in advanced myxoid liposarcoma patients[34]. Until today, nine different types of FUS/DDIT3 fusion genes have been described, involving predominantly the central and C-terminal parts of the FUS-gene and nearly always the whole DDIT3 gene[22]. We describe here for the first time a new fusion type (Figure 1) including the RNA binding domain of the FUS gene, which is not found in the other fusion types except for type 8. Whether this new rare fusion gene will be translated to a protein or will have any promoting effect on tumor development is not clear and is hard to study due to the rarity of these variants. We found no differences between the type of FUS/DDIT3 fusion gene and kinases activated. Till now, the molecular variability of fusion types has not shown to have any effect on transforming capacities, adipogenesis nor prognosis in myxoid liposarcoma[5,35].

We showed that kinases associated with NF-kappaB pathway were highly active in myxoid liposarcoma. In the atypical (IKK independent) NF-kappaB pathway, phosphorylation of inhibitors of NF-kappaB (IkB), and subsequent activation of NF-kappaB (p65) is controlled by casein kinase 2 and tyrosine kinase-dependent pathways (figure 5)[36,37]. We did not measure NF-kappaB pathway activation by analysis of downstream products or electrophoretic mobility shift assays. Göransson *et al. *has recently *s*hown that NF-kappaB is a major factor controlling IL8 transcription in FUS-DDIT3 expressing cells. This could be explained by direct binding of FUS/DDIT3 to the C/EBP-NF-kappaB composite site of the immediate promoter region of IL8. Moreover, FUS/DDIT3-GFP expressing cell lines showed upregulation of the NF-kappaB controlled genes *LCN2 *and *MMP1 *whereas DDIT3 had little effect. These findings were also quantitatively confirmed by RT-PCR[17]. Active (phosphorylated) p65 was present in cell lysates of myxoid liposarcoma cell cultures and cell lines. We did not explicitly show that the phosphorylated p65 protein was located in the nucleus/nuclear fraction. Phosphorylation of p65 could be counteracted by TBB, an inhibitor of the casein kinase 2 and resulted in decreased cell viability as shown in figure 3 and 4. This suggests that NF-kappaB signaling is active in myxoid liposarcoma and that its activation is, at least in part, regulated via the atypical pathway. This is an important finding which suggests that NF-kappaB pathway inhibition might be beneficial in myxoid liposarcoma patients with advanced disease.

The exact driving force behind NF-kappaB activation in myxoid liposarcoma is unclear. Gene expression studies revealed that p50 was significantly upregulated in FUS/DDIT3 transfected fibroblastic cell lines[38]. This suggests that NF-kappaB (p50) transcription in myxoid liposarcoma might be regulated by the FUS/DDIT3 fusion gene. After translocation to the nucleus, transcriptional activation of NF-kappaB requires multiple co-activating proteins[39]. The C-terminus of FUS co-activates p65 and plays a pivotal role in NF-kappaB mediated transcription though this C-terminus is lost in the FUS/DDIT3 fusion protein. Recent studies showed that the FUS/DDIT3 fusion protein facilitates NF-kappaB binding to its target genes, probably in an indirect manner[19,39-41]. The FUS-DDIT3 fusion protein deregulates NF-kappaB controlled genes by interaction with nuclear factor of kappa light polypeptide gene enhancer in B-cells inhibitor zeta (NFKBIZ)[19]. This synergistic role between a fusion protein and activation of NF-kappaB signaling might also be important in other translocation based sarcomas and has already been shown in Bcr-Abl mediated leukemias[42].

In all myxoid liposarcoma samples we showed overexpression of casein kinase 2, which has been shown in many other neoplasms[43]. We showed inhibition of casein kinase 2 and subsequent decreased levels of active p65 to be associated with decreased viability and increase in caspase 3 protein expression in myxoid liposarcoma cells. Caspase 3 is released by cleavage of its inactive precursor procaspase 3, and mediates apoptosis[44,45]. Decreased cell viability with increased levels of the effector caspase 3 therefore suggests caspase 3 mediated apoptosis. Recently, phase I trials have been started to test the effect of casein kinase 2 inhibitors *in vivo *which seems to be promising[46].

In addition to kinases associated with NF-kappaB, Fyn, Lck and Yes were most active as indicated by specific sequences on the chip. They are members of the Src family of kinases. Src plays an important role in embryonic development, cell growth and cell survival and activating mutations in Src have been reported in colorectal carcinoma[47,48]. Src signaling can lead to downstream activation of ERK/MAPK and PI3K/AKT signaling. Activation of both pathways in myxoid liposarcoma is associated with more aggressive behavior[49]. The Src pathway can be inhibited by the small molecule tyrosine kinase inhibitor dasatinib limiting cell growth in various cancers *in vitro*, thereby having promising therapeutic potential[16,50,51]. Immunoblotting confirmed the expression of Src and phosphorylation of Src at Y419 in myxoid liposarcoma cell cultures and cell lines. Dasatinib treatment showed a reduction in phosphorylated (active) Src and a decrease in cell viability. However, this latter effect was only very mild with maximum decrease in viability of only 40% maximally, and no IC50 levels could be calculated. This might be explained by Src pathway activation occurring upstream, close to its receptor (figure 5) and that the effect of the inhibition of Src phosphorylation might be (partly) circumvented by crosstalk activation downstream. Our data suggest that the active Src pathway is not crucial for myxoid liposarcoma survival and that monotherapy with dasatinib is no suitable option for treatment, although the additional effect of dasatinib *in vivo *through inhibition of angiogenesis is not encountered here.

Combinations of different drugs (including dasatinib) have been shown to act synergistically in many tumors and combination drug therapy is commonly used in cancer treatment[50]. Recently, a synergistic effect of dasatinib when combined with other drugs (i.e. oxaliplatin) has been described in colorectal carcinoma[50]. Since we showed NF-kappaB and Src to be the two most active pathways we studied the effect of combination of dasatinib and TBB and we found a enhanced effect on cell viability of myxoid liposarcoma cells *in vitro*. To be more specific: L1357 cells show 80% viability at maximum dasatinib dose (5000 nM), whereas viability was only 5% at lower concentration of dasatinib (500 nM) at IC50 for TBB (figure 3). However, it was not possible to calculate if this enhancement was also a true synergistic effect as IC50 values for dasatinib could not be calculated (figure 3)[52]. IC50 values for TBB (but not for dasatinib) could be calculated for most primary cultures and cell lines, but not for L1187 and L1434. Though cell line 1765-92 responded well to TBB treatment, no enhancement could be observed upon addition of dasatinib, which might be related to a relative resistance of 1765-92 cells to dasatinib as also visible from figure 3A. Future experiments, for instance studying the changes at the kinome level upon dasatinib treatment may reveal (1) why dasatinib is not effective as a monotherapy but is effective in combination with TBB, and (2) what might be the exact underlying mechanism why 1765-92 myxoid liposarcoma cells showed resistance for dasatinib treatment and thereby the absence of enhancement in combination treatment as was observed for the other cell line and primary cultures.

## Conclusion

In conclusion our results indicate that the NF-kappaB and Src pathway include the most active kinases in myxoid liposarcoma, and inhibition of casein kinase 2 and thereby interference with kinases associated with the NF-kappaB pathway decreases cell viability *in vitro*, the effect of which can be enhanced by inhibiting src- signalling using dasatinib.

## Methods

### Reagents

Dasatinib (Sprycel, BMS- 354825) was obtained from Bristol-Myers Squibb (New York, USA) and TBB from Calbiochem (San Diego, CA). Both drugs were dissolved in Dimethylsulfoxide (DMSO).

### Cell cultures and cell lines

The two myxoid liposarcoma cell lines 402-91 and 1765-92, and gastro-intestinal stroma cell tumor cell line (GIST882) were kindly provided by Prof. Dr. P. Aman (Lundberg Laboratory for Cancer Research, Department of Pathology, Göteborg University, Goteburg, Sweden) and Prof. Dr. J. Fletcher (Brigham and Women's Hospital, Boston, USA) respectively[53,54]. Jurkat and HeLa cell lines (American Type Culture Collection, Rockville, MD) were used as positive controls for Western blotting. Myxoid liposarcoma cell lines, primary cultures of four myxoid liposarcomas (L1187, L1357, L1434 and L2187) and two cell cultures of normal bone marrow derived mesenchymal stem cells (L2361 and L2370) were cultured in RPMI 1640 (Gibco, Invitrogen Life-Technologies, Scotland, UK), supplemented with 10% heat-inactivated fetal calf serum (Gibco). Cells were grown in a humidified incubator at 37°C with 5% CO2. In addition, two samples (402-19 and L1357) were analyzed after also culturing in starved RPMI 1640, containing 0,5% fetal calf serum.

### RT-PCR and karyotyping

Diagnosis of the primary tumors from which the cultures were obtained was performed on histology. Primary tumors were analyzed for their tumor specific translocation with double-fusion fluorescence in situ hybridization (FISH) and cell lines were karyotyped with Combined Binary Ratio Labeling (COBRA) as previously described [55-57]. In primary cultures, tumor cells were genotyped for the presence of the fusion gene by RT-PCR. Total RNA was isolated using TRIzol (Invitrogen, Breda, The Netherlands). Complementary DNA was synthesized from 1 μg of total RNA using oligo dT primers and Superscript II MMLV reverse transcriptase (Life Technologies, Carlsbad, CA). Reverse-transcription polymerase chain reaction (RT-PCR), sample purification and DNA sequence analysis were performed as described previously [58]. The following primers were used: *FUS*-forward, CAG AGC TCC CAA TCG TCT TAC GG and *DDIT3*-reverse, GAG AAA GGC AAT GAC TCA GCT GCC.

### Kinome array analysis

Kinase substrate peptide arrays (Pepchip Kinomics, Pepscan Presto, Lelystad, the Netherlands) containing 1024 different kinase substrates spotted in triplicate together with 16 negative, and 16 positive controls were used and successfully used in prior studies [16,59]. The distribution of the target sequences in terms of kinase recognition is described in detail on the website http://www.pepscanpresto.com/index.php?id=30. Cells were harvested during their exponential growth phase and lysated as previously described. Concentration of the protein lysates was measured using the *DC *Protein Assay (Biorad, Hercules, CA, USA). Analysis was performed as described earlier, including the two serum-starved samples[16]. Autoradiographic signals were sensed by phosphoimage screen and scanned by Typhoon 9400 phosphoimager (GE Healthcare, Piscataway, NJ). At least 1 × 10^6 ^hits were collected.

### Data analysis

The scanned images were analyzed and quantified using ImageQuant software (Molecular Dynamics, Sunnyvale, CA). For further data mining R-packages Affyio and Limma were used http://www.bioconductor.org. Quality of the triplicates and distribution of the data was assessed and quartile normalization (Affyio) was performed as previously described[16]. Median intensities of the triplicates were calculated and the top 100 spots were imported for core analysis in Ingenuity Pathway Analysis (IPA, Ingenuity Systems, http://www.ingenuity.com). IPA is a literature based program that calculates the probability of involvement of identifiers, in this case combinations of kinases, in 74 different pathways. Data of the myxoid liposarcoma cell lines and cultures were averaged to find the common denominators that are active in all cultures[16]. To ensure that artificially induced kinase activity due to cell culturing interfered with tumor specific kinase activity, the same analysis was run excluding cell cycle related kinases as well as after starvation. Specificity of activated kinases and activated pathways in myxoid liposarcoma was verified by comparison the same analysis of four colorectal carcinoma cell lines and thirteen chondrosarcoma cell lines and cultures using Limma[16].

### Immunoblotting

Western blotting was performed as previously described[58]. Rabbit polyclonal antibody to phosphorylated Src (Y419) was obtained from R&D Systems (1/2000; Minneapolis, MN USA). Monoclonal antibody to total Src and alpha-tubulin were obtained from Upstate Biotechnology (clone GD11, 1/2000, Lake Placid, NY, USA) and Sigma Aldrich (St. Louise, MO, USA), respectively. Rabbit polyclonal antibodies against casein kinase 2alpha; NF-kappaB p65, phospho- NF-kappaB p65 (S468) and caspase 3 were obtained from Cell Signaling Technology (Beverly, MA). HeLa cell lines, untreated and treated with TNFalpha (20 ng/ml) were used as a positive control for casein kinase 2alpha and NF-kappaB p65/phospho- NF-kappaB p65, respectively, according to the manufacturer's protocol.

### In vitro viability assays

Measurement of metabolic activity by a WST-1 colorimetric assay (Roche Diagnostics GmbH, Penzberg, Germany) was used as a read-out system for cell viability in response to kinase inhibitors. Dasatinib was used to inhibit Src-pathway; TBB was used to inhibit casein kinase 2, which is an important kinase in atypical NF-kappaB signalling. After harvesting, 2000 cells/well of every cell line and primary culture were seeded into 96-well flat-bottom plates. After 24 hours, increasing concentrations of the drugs (50, 100, 200, 500, 1000 and 5000 nM for dasatinib and 10, 20, 50, 100, 200 and 500 μM for TBB respectively) were added or 0,1% DMSO as vehicle control, each condition in quadruplicate. Ten percent serum supplementation was used for all experiments. After 3 days of treatment, absorbance was measured on a Victor Multilabel Counter 1420-042 (Perkin Elmer, Groningen, The Netherlands) at 450 nm, and was corrected for background and averaged. GIST882 and Jurkat cell lines were used as positive controls for dasatinib and TBB experiments, respectively[16]y. In combination experiments, 2000 cells were plated overnight followed by treatment with dasatinib which was added 30 minutes after TBB administration. In these experiments, increasing concentrations of dasatinib at IC50 concentrations of TBB were used.

## Competing interests

The authors declare that they have no competing interests.

## Authors' contributions

SMW carried out kinome studies, immunoblotting, *in vitro *studies and DNA sequencing and drafted the manuscript. YMS participated in design of the kinome assay, immunoblotting and statistical analysis. IHB participated in kinome studies and immunoblotting. KS carried out the karyotyping, analysis of DNA sequences and participated in design of cell culturing. PCWH participated in design and coordination and helped to draft the manuscript. JVMGB designed and supervised the study and helped to draft the manuscript. All authors read and approved the final manuscript.

## Supplementary Material

Additional file 1 **Top 100 of activated kinases in MSCs obtained by kinome analysis**. Table shows list of kinases found to be activated in mesenchymal stem cells, in decreasing order. The intensity correlates with the radioactivity of ^33^P incorporated in the substrates by their active kinases. The number of hits are the number of substrates related to the respective kinase. Click here for file
