# Supplementary figures and images for: Kinome profiling of myxoid liposarcoma reveals NF-kappaB-pathway kinase activity and Casein Kinase II inhibition as a potential treatment option
Source: Mol Cancer. 2010 Sep 23;9:257. doi: 10.1186/1476-4598-9-257 (PMC2955617; doi:10.1186/1476-4598-9-257)

**Additional file 1:**

**Title: Top 100 of activated kinases in MSCs obtained by kinome analysis**


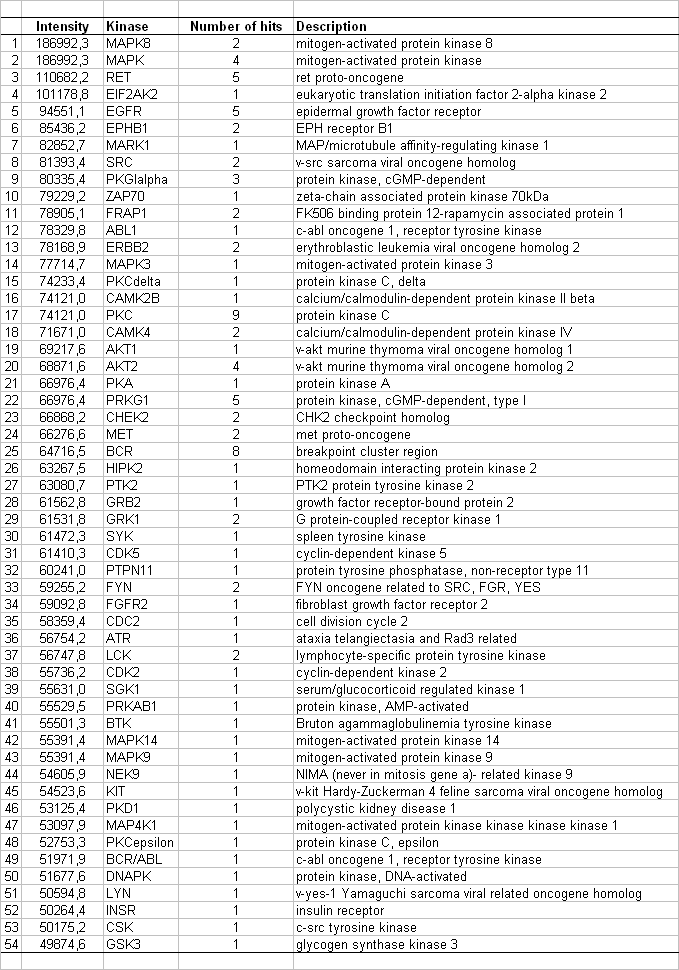

Supplement: Additional file 1 — Top 100 of activated kinases in MSCs obtained by kinome analysis. Table shows list of kinases found to be activated in mesenchymal stem cells, in decreasing order. The intensity correlates with the radioactivity of 33P incorporated in the substrates by their active kinases. The number of hits are the number of substrates related to the respective kinase. [file 1476-4598-9-257-S1.DOC]
